# Selective Cytotoxic Activity of Prodigiosin@halloysite Nanoformulation

**DOI:** 10.3389/fbioe.2020.00424

**Published:** 2020-05-26

**Authors:** Ivan Guryanov, Ekaterina Naumenko, Farida Akhatova, Giuseppe Lazzara, Giuseppe Cavallaro, Läysän Nigamatzyanova, Rawil Fakhrullin

**Affiliations:** ^1^Institute of Fundamental Medicine and Biology, Kazan Federal University, Kazan, Russia; ^2^Dipartimento di Fisica e Chimica, Università degli Studi di Palermo, Palermo, Italy; ^3^Consorzio Interuniversitario Nazionale per la Scienza e Tecnologia dei Materiali, INSTM, Florence, Italy

**Keywords:** cancer, anti-cancer drugs, comet assay, drug delivery, genotoxic effect, halloysite nanotubes, malignant cells, prodigiosin

## Abstract

Prodigiosin, a bioactive secondary metabolite produced by *Serratia marcescens*, is an effective proapoptotic agent against various cancer cell lines, with little or no toxicity toward normal cells. The hydrophobicity of prodigiosin limits its use for medical and biotechnological applications, these limitations, however, can be overcome by using nanoscale drug carriers, resulting in promising formulations for target delivery systems with great potential for anticancer therapy. Here we report on prodigiosin-loaded halloysite-based nanoformulation and its effects on viability of malignant and non-malignant cells. We have found that prodigiosin-loaded halloysite nanotubes inhibit human epithelial colorectal adenocarcinoma (Caco-2) and human colon carcinoma (HCT116) cells proliferative activity. After treatment of Caco-2 cells with prodigiosin-loaded halloysite nanotubes, we have observed a disorganization of the F-actin structure. Comparison of this effects on malignant (Caco-2, HCT116) and non-malignant (MSC, HSF) cells suggests the selective cytotoxic and genotoxic activity of prodigiosin-HNTs nanoformulation.

## Introduction

Currently, the number of effective chemotherapeutic agents for the therapy of neoplasms is very limited (Patel et al., 2010). Existing drugs often have toxic side effects on adjacent non-malignant cells and tissues. The problem of safety of the medications used in chemotherapy is of pivotal importance, one of the ways to solve it is the search for non-toxic substances and fabrication of new drug formulations. Natural drugs that can suppress the proliferation of cancer cells and metastasis formation are becoming increasingly popular (Huryn and Wipf, 2014). Prodigiosin, a bioactive secondary metabolite produced by *Serratia marcescens* and certain other bacteria is of particular interest (Williamson et al., 2006). The antitumor properties of prodigiosin have already been confirmed in previous studies (Montaner and Perez-Tomas, 2003; Perez-Tomas et al., 2003; Francisco et al., 2007; Lins et al., 2015). Prodigiosin can induce apoptosis in hematopoietic, colorectal, gastric cancer cells (Montaner and Perez-Tomas, 2003), human breast carcinoma cell lines (Lu et al., 2012), choriocarcinoma (Zhao et al., 2019) and prostate cancer cell lines (PC3) *in vitro* and JEG3 and PC3 tumor-bearing nude mice *in vivo* (Li et al., 2018) with metastases suppression (Zhang et al., 2005). The cytotoxic activity of prodigiosin against various human cancer cell lines and relatively lower toxicity toward non-malignant cells has been demonstrated previously (Francisco et al., 2007; Stankovic et al., 2014; Zhao et al., 2019). In addition, prodigiosin can be used to replace synthetic colorants in food industry and sunscreen cosmetics (Darshan and Manonmani, 2015).

Hydrophobic nature of prodigiosin is an obvious disadvantage for medical and biotechnology applications. Limited aqueous solubility of prodigiosin result in poor absorption and low bioavailability (Tran et al., 2019), as well as it may disturb regular distribution of prodigiosin in biological fluids. Bioavailability of prodigiosin can be enhanced similarly, as reported previously for anticancer drug doxorubicin (Li et al., 2017) and curcumin (Ni et al., 2019), employing fabrication of nanoscale drug formulations to overcome the limitations caused by the intrinsic hydrophobicity prodigiosin. Recently, a technique of prodigiosin encapsulation was developed and anticancer effect of targeted nanoformulations of prodigiosin was investigated (Zhao et al., 2019).

Targeted delivery and controlled release of antitumor drugs, antibiotics, enzymes, and nucleic acids are currently among of the most significant challenges in biomedicine (Martín del Valle et al., 2009; Tiwari et al., 2012; Yendluri et al., 2017). The pharmacokinetics and pharmacodynamics of a number of drugs require special procedures for their administration. Using nanoscale drug delivery vehicles is one of the most promising approaches for targeted drug delivery systems (Miyazaki and Islam, 2007). Nanocarrier-based drugs allow preventing possible side effects of drugs and to overcome physiological barriers of the body (for example, blood–brain barrier) (De Jong and Borm, 2008). Nanoscale anticancer formulations can be designed using natural substances or derivatives, such as chitosan, dextran, gelatin, alginate, liposomes (De Jong and Borm, 2008), gold (Kohout et al., 2018; Singh et al., 2018) and magnetic iron oxide nanoparticles (Dulińska-Litewka et al., 2019; Rozhina et al., 2019), mesoporous silica nanoparticles (Li et al., 2019), carbon nanotubes (Cirillo et al., 2019) and clay nanotubes (Naumenko and Fakhrullin, 2017, 2019; Yendluri et al., 2017). Natural aluminosilicate halloysite, due to its tubular structure and surface chemistry, is a potent platform to fabricate nanocontainers for drug-delivery systems. Halloysite has a hollow tubular structure, with the length of up to 1 μm, external diameter 70 nm and an inner lumen 15 nm (Shchukin et al., 2005). Halloysite nanotubes are widely used for the fabrication of polymeric nanocomposites to enhance their tensile strength and stability (Naumenko et al., 2016; Suner et al., 2019). The tubular structure of halloysite allows the internal cavity to be loaded with various macromolecules including drugs, proteins, and nucleic acids, followed by the release of the loaded compounds in the delivery region (Joussein et al., 2005). Such features as very low toxicity (Lai et al., 2013; Fakhrullina et al., 2015) and directed modification of the surface and internal cavity (Abdullayev et al., 2012; Tarasova et al., 2019; Rozhina et al., 2020) make halloysite nanotubes promising candidates for the fabrication of nanocontainers for theranostic targeted drug delivery (Hu et al., 2017). Halloysite nanotubes can be efficiently filled with hydrophobic drug via physical entrapping in the internal cavity (Naumenko and Fakhrullin, 2017, 2019; Fakhrullina et al., 2019). As a result, halloysite-based drug formulation demonstrate lower drug side effects, render the protection of drug molecules from possible degradation in aggressive conditions (low/high pH, enzymatic activity), increase the aqueous solubility of hydrophobic insoluble drugs, accumulate in pathological sites in the body, and help controlling drug release rates (De Jong and Borm, 2008; Naumenko and Fakhrullin, 2017, 2019).

In this paper we report for the first time fabrication of prodigiosin-based nanoformulation (p-HNTs) and its effects on viability of malignant and non-malignant cells.

## Materials and Methods

### Prodigiosin-HNTs Fabrication and Characteristics

The red pigment prodigiosin was obtained by cultivation of the producer strain *S. marcescens ATCC 9986* on agarized peptone–glycerol medium. Pigment purification was performed as described previously (Guryanov et al., 2013). Ethanol solution (96% vol. 300 μl of purified prodigiosin (4.4 μg) was mixed with glycerol (70 μL), dry HNTs (30 mg) in centrifuge tube and placed into desiccator for loading by vacuum displacement (Supplementary Figure S1). Prodigiosin loading procedure was performed for 24 h. Subsequently, the loading efficiency was evaluated by thermogravimetric analysis (TGA) while Fourier transform infrared spectroscopy (FT-IR) highlighted the interaction mechanism and involved functional groups. Optical absorption spectra of purified prodigiosin in ethanol and extracts of glycerol-HNTs and prodigiosin-HNTs after 30 min and 2 h exposure in PBS were obtained and compared for estimation of pigment release from loaded halloysite nanotubes. Absorption spectra were analyzed using a Lambda 35 spectrometer (PerkinElmer).

### Dark-Field Imaging and Hyperspectral Microscopy

Dark-field images and reflected light spectra were obtained using an Olympus BX51 (Olympus) upright microscope equipped with a CytoViva^®^ enhanced dark-field condenser with a halogen light source (150 W) Fibre-Lite DC-950 (Dolan-Jener) and control module ProScan III (JH Technologies). Images were obtained using acquisition software for visualization Exponent 7 (Dage-MTI). Spectra were registered using a Specim V10E spectrometer and CCD camera in the range between 400 and 1000 nm with a spectral resolution –2 nm. Hyperspectral data were collected with ENVI software, version 4.8 (Harris Geospatial Solutions) (Akhatova et al., 2018). The presence of prodigiosin inside the treated cells was visualized using a CytoViva dual fluorescence module. Images were processed using ImageJ freeware (NIH). The cells were fixed on coverslips, nuclei of the cells were stained with DAPI. An X-cite 120Q wide-field fluorescence microscope excitation light source (Excelitas Technologies) and CytoViva^®^ Dual Mode Fluorescence system equipped with the Triple Pass Filter were used to image DAPI nuclear staining with transmitted fluorescence illumination imaging, exposure time was 100 μs. Fluorescence nuclei images were rendered with artificial red color to enhance local contrast using GIMP software, version 2.10.8. The resulting dark-field images were merged with transmission fluorescence images using the freely available image processing GIMP.

### Fourier Transform Infrared Spectroscopy

Fourier transform infrared spectra were registered using a Frontier FTIR spectrometer (PerkinElmer). The measurements were conducted on KBr pellets at room temperature in the range between 500 and 4000 cm^–1^ with a spectral resolution of 2 cm^–1^.

### Thermogravimetry

Thermogravimetry (TG) experiments were carried out by means of a Q5000 IR apparatus (TA Instruments) under the nitrogen flows of 25 cm^3^ min^–1^ for the sample and 10 cm^3^ min^–1^ for the balance. The mass of each sample was ca. 5 mg. TG measurements were conducted between 25 and 600°C using a constant heating rate of 20°C min^–1^. The temperature calibration was carried out by means of the Curie temperatures of standards (nickel, cobalt, and their alloys) (Blanco et al., 2014). The encapsulation efficiency into HNTs was determined by considering the rule of mixtures for the residual mass at 600°C. Details are provided in literature (Lisuzzo et al., 2019).

### Atomic Force Microscopy

Atomic force microscopy (AFM) images of HNTs and cells were made using a Dimension Icon microscope (Bruker) operating in PeakForce Tapping mode. ScanAsyst-air (Bruker) probes were used to obtain images (nominal length 115 μm, tip with a radius of 2 nm, spring stiffness 0.4 N m^–1^). Images were obtained at 512–1024 scan lines at a scanning speed of 0.8–0.9 Hz. The adhesion of the nanoparticles was analyzed using an atomic force microscope and calculated from 30 × 30 nm sites on the nanoparticles surface. The obtained data were processed using Nanoscope Analysis software version 1.7 (Bruker).

### Cell Culture

A frozen stock of HCT116, Caco-2, rat adipose-derived MSCs and human skin fibroblasts (HSF) was grown for 7 days in α-MEM (Sigma-Aldrich) supplemented with 10% of fetal bovine serum (Thermo Fisher Scientific), 100 IU/ml penicillin, 100 μg/ml streptomycin and 2 mM L-glutamine on 25 cm^2^ tissue culture flasks in humidified atmosphere with 5% CO_2_ at 37°C. After cultivation, cells (2.5 × 10^5^) were seeded on 24 well cell culture plates (BD Biosciences) and cultured for 48 h in CO_2_ incubator at 37°C. Glycerol-HNTs or prodigiosin-HNTs in PBS were added to each well to final concentration 100 μg per ml and cells were incubated for 48 h.

### Comet Assay (Alkaline Comet Assay)

Live trypsinized cells were mixed with low-melting agarose (1.5%) and added to fully frosted slides precoated with 1% normal melting point agarose. After solidification, the slides were lysed in buffer and alkaline solutions (lysis buffer pH 10, alkaline solution pH 13, Tris-acetate-EDTA buffer 2 h at 4°C. After lysis, the slides were immersed in the neutralizing solution (0.4 M Tris, pH 7.5) for 15 min. Then the slides were placed in the alkaline solution (300 mM NaOH, 1 mM EDTA-Na_2_, pH 13) for 20 min to allow DNA unwinding and subsequently electrophoresed for 30 min at 20 V, 300 mA. Upon completion of the electrophoresis the slides were placed in 70% ethyl alcohol for 5 min at room temperature, for DNA fixation. After drying at room temperature for 1 h the slides were stored in a dry and dark place until further analysis. Finally, the slides were stained with ethidium bromide and then visualized using confocal microscopy (Carl Zeiss LSM 780) equipped with diode laser (405nm), argon laser (488 nm) and He-Ne laser (633nm). Hundred randomly captured nuclei were examined from each slide in two independent experiments. The analysis does not include comet DNA of apoptotic cells detected on microscopic preparations as fluorescent comets with a broad diffuse tail and a very small head, so-called hedgehogs. Data was processed using CometScore software (v. 2.0).

### Cytoskeleton Visualization

F-actin was stained with Alexa Fluor 488^®^ conjugate of phalloidin according to the protocol, provided by Life Technologies, nuclei were stained with DAPI. Samples were visualized using confocal microscopy. Images were processed using ZEN software.

### Live/Dead Staining of Cell Cultures

Viability of cells was tested using Cell Viability Imaging Kit (Blue/Green) (Life Technologies) according to the protocol, provided by producer. Samples were using confocal microscopy. Living cells were visualized as blue, and dead cells as green.

## Results and Discussion

We have isolated prodigiosin by acidic ethanol extraction from *S. marcescens* bacteria and purified as described previously (Guryanov et al., 2013). Prodigiosin purity was confirmed using UV–vis spectroscopy (Supplementary Figure S1). Next, prodigiosin was loaded (dissolved in ethanol/glycerol solvent) into the lumens of halloysite nanotubes via vacuum-facilitated loading (Dzamukova et al., 2015), as schematically shown in Supplementary Figure S2. To determine the stability of prodigiosin loaded halloysite nanotubes we investigated prodigiosin leakage in phosphate buffered saline (PBS) at 37°C (Supplementary Figure S1). Absorption spectra of p-HNTs suspension in PBS confirm the prodigiosin binding to halloysite. Spectrophotometry data demonstrate non-specific prodigiosin absorption, the increase of absorption value over time in this experiment can be explained by the following dissociation of hydrophilic HNTs in PBS solution. Spectral signatures of p-HNTs obtained in reflected light mode demonstrate the effective loading of prodigiosin due to the presence of characteristic peaks of pure HNTs and prodigiosin in complex spectra (Supplementary Figure S3).

**FIGURE 1:**
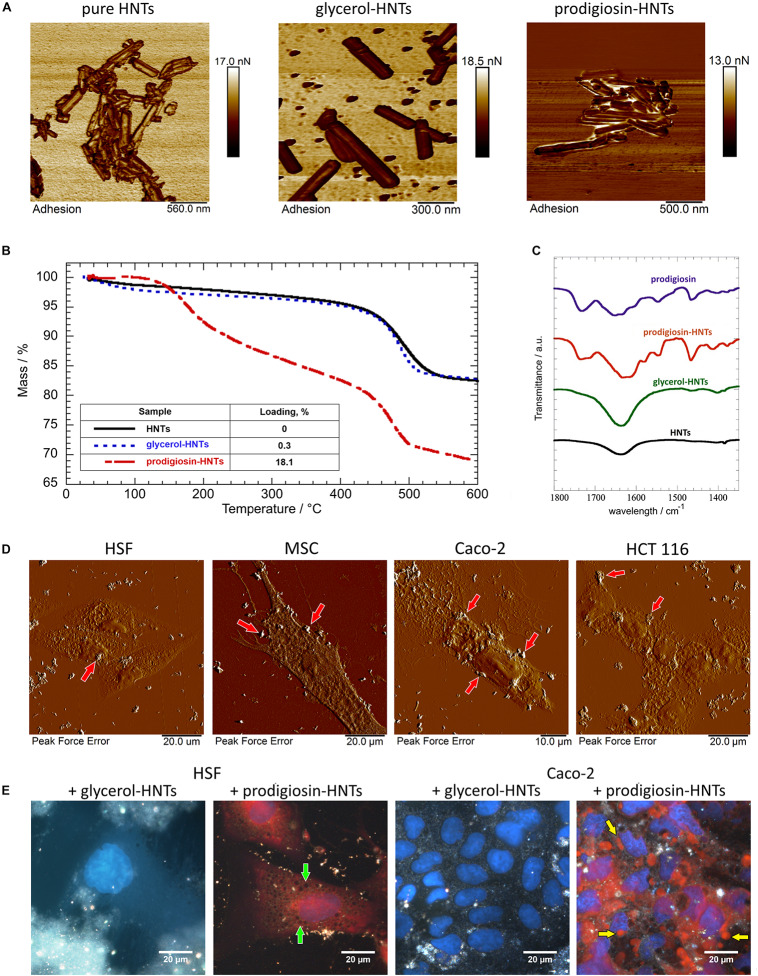
Characterization of prodigiosin-HNTs. Atomic force microscopy images **(A)** of pure HNTs and loaded with glycerol and prodigiosin, demonstrating the change of nanotubes surfaces adhesiveness. TGA analysis **(B)** of prodigiosin-loaded HNTs demonstrated that this complex contains 18% of prodigiosin. FT-IR spectra **(C)** in the wavelength range between 1350 and 1800 cm^–1^. AFM images of the cells **(D)** indicate that hydrophobic prodigiosin-HNTs strongly attach to the cell membrane, forming large clusters. **(E)** prodigiosin exhibits autofluorescence, therefore its leakage from the nanotubes and the presence in the cytoplasm can be visualized using fluorescence module of hyperspectral microscope CytoViva^®^. More details see in Supplementary Figure S4.

The detailed characterization of HNTs results are shown in Figure 1. Prodigiosin, as a hydrophobic compound (de Araújo et al., 2010), after the loading into halloysite can located within halloysite lumen and on the surface of the tubes (Figure 1A). This, in turn, increases the adhesion of prodigiosin-loaded halloysite nanotubes. AFM images of halloysite nanotubes loaded with glycerol and prodigiosin (Figure 1A) confirm the increase of non-specific adhesion activity after prodigiosin loading (10.5 ± 0.7 nN) compared with the control pristine halloysite nanotubes (4.5 ± 0.6 nN). We also observe a slight increase in surface adhesion of nanotubes with glycerol (7.3 ± 0.5 nN). We suggest that an increase in the adhesion of the surface of the p-HNTs may contribute to their aggregation.

The thermal behavior of modified HNTs (glycerol-HNTs and prodigiosin-HNTs) was investigated by thermogravimetric method as described previously (Blanco et al., 2014). As evidenced in Figure 1B both glycerol-HNTs and p-HNTs exhibit a mass loss in the temperature interval between 450°C and 550°C. These results highlighted that the presence of the interlayer water molecules of halloysite is preserved in the functionalized HNTs. Moreover, p-HNTs showed an additional degradation step in the temperature range between 180 and 320°C that can be attributed due to the destabilization of bonds in the prodigiosin molecule (Sumathi et al., 2014). The corresponding mass loss was estimated at 8.73 wt%. According to literature, the mass loss between 25°C and 120°C (ML_120_) can be ascribed to the moisture content of the investigated material (Cavallaro et al., 2018). Table 1 evidences that the addition of glycerol generated an enhancement of the water amount physically adsorbed on HNTs, while the opposite effect was detected after the introduction of prodigiosin in the halloysite nanostructure. Further insights on the functionalization of HNTs surfaces were obtained by comparing the residual masses at 600°C (MR_600_). We estimated similar MR_600_ values for HNTs and glycerol-HNTs. By contrast, the presence of prodigiosin induced a significant MR_600_ reduction indicating the successful modification of halloysite.

**TABLE 1:** Thermogravimetric parameters for HNTs, glycerol-HNTs and prodigiosin-HNTs.

Material	ML_150_/wt%	MR_600_/wt%
HNTs	1.30	82.3
Glycerol-HNTs	2.26	82.9
Prodigiosin-HNTs	0.707	68.7

Figure 1 compares the FT-IR spectra of modified HNTs (glycerol-HNTs and prodigiosin-HNTs) with those of pristine HNTs and prodigiosin (Figure 1C). The wavelength range between 1350 cm^–1^ and 1800 cm^–1^ can be considered as a fingerprint region for prodigiosin, which presents numerous characteristic signals that were not detected neither for pure HNTs nor for glycerol-HNTs. On the other hand, the typical FT-IR peaks of the drug at 1467 cm^–1^ (bending of C–H) and 1548 cm^–1^ (aromatic C=C, NO_2_ stretch) (Suryawanshi et al., 2014) were observed in the p-HNTs confirming the successful loading. Interestingly, the band at 1734 cm^–1^ (C=O stretching vibration) (Arivizhivendhan et al., 2015) of prodigiosin was split in two peaks (1734 and 1717 cm^–1^) in the p-HNTs. According to the literature (Cavallaro et al., 2012), this result could indicate that the delocalization of the negative charge along the carboxylate group of the drug is no longer present in the loaded HNTs. On this basis, the loading of prodigiosin in HNTs might be partly related to electrostatic interactions between the two components.

**FIGURE 2:**
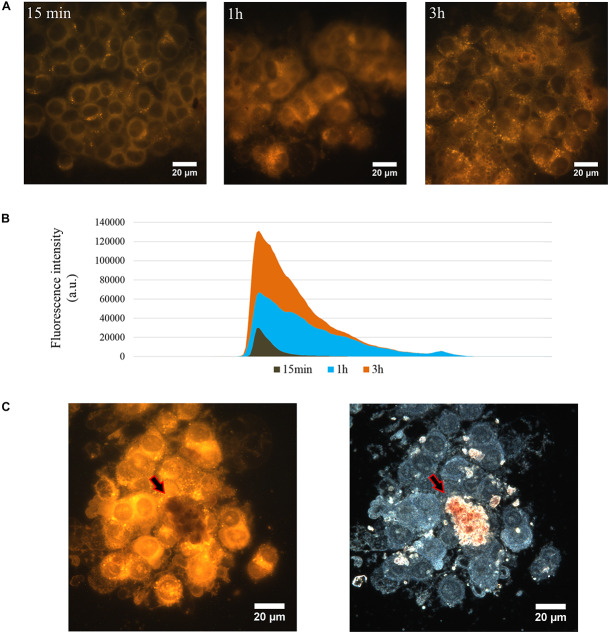
The release of prodigiosin from p-HNTs in cytoplasm of HCT116 cells. Fluorescence images of p-HNTs-treated cells were taken after 15 min, 1 and 3 h of incubation **(A)**. fluorescence intensity histogram **(B)** demonstrated that the fluorescence intensity increased proportionally to the incubation time. Comparative analysis of dark-field and fluorescencee images **(C)** demonstrates the absence of extracellular leakage of prodigiosin. The arrows indicate the red colored p-HNTs aggregate on the cell surface which cannot be visualized on fluorescent image.

**FIGURE 3:**
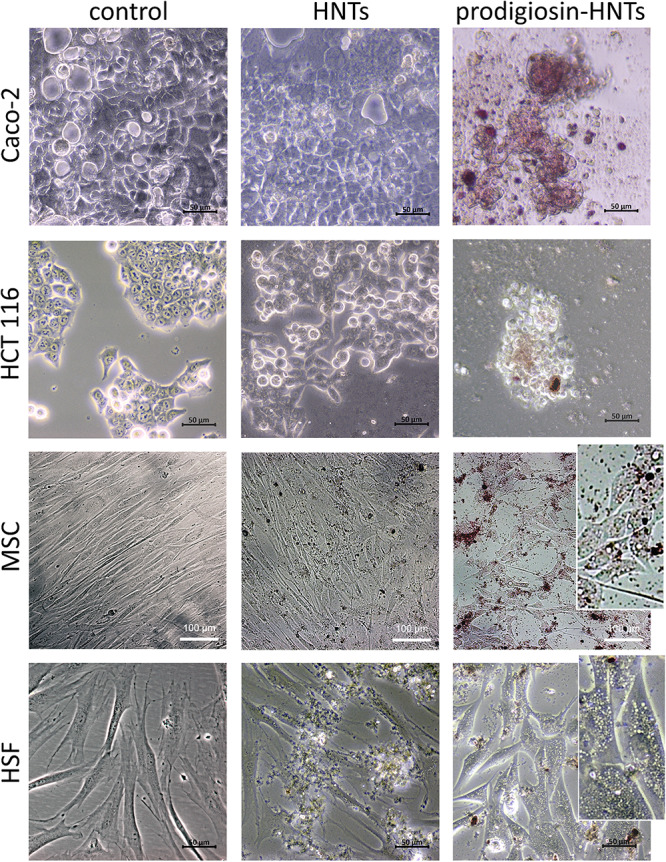
Comparison of the effect of halloysite nanotubes loaded with glycerol (HNTs) and prodigiosin-HNTs on malignant (Caco-2, HCT116) and non-malignant cells (MSC, HSF). All cultures were grown in Minimum Essential Medium Eagle – Alpha Modification (α-MEM) with 5% CO_2_ in air and humidity at 37°C. The addition of prodigiosin-HNTs during the cultivation of cancer cells (Caco-2, HCT116) led to aberration of their morphology and subsequent detachment from the surface of the dish (Caco-2/prodigiosin-HNTs, HCT116/prodigiosin-HNTs). Glycerol-loaded HNTs which were used as a control did not affect the morphology and viability of all cell types. The presence of prodigiosin-HNTs in the culture medium of non-malignant cells increased the cytoplasm vacuolization of some cells (MSC/prodigiosin-HNTs and HSF/prodigiosin-HNTs inserts).

**FIGURE 4:**
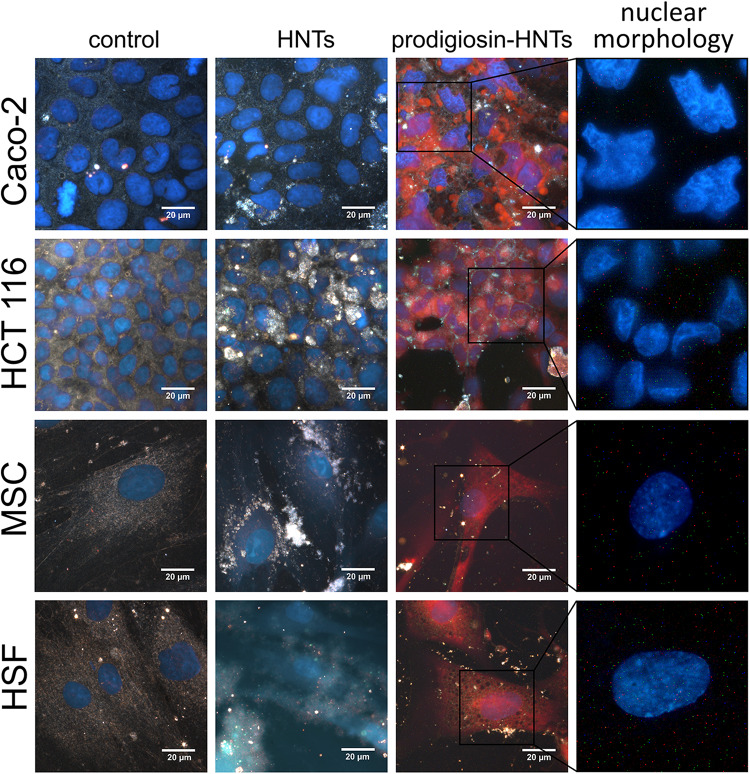
Hyperspectral images for comparison of the effect of halloysite nanotubes loaded with glycerol (HNTs) and prodigiosin-HNTs on malignant (Caco-2, HCT 116) and non-malignant cells (MSC, HSF). All cultures were grown in α-MEM with 5% CO_2_ in air and humidity at 37°C. Nuclei (blue) were labeled with DAPI. Purple non-specific staining of the cells cytoplasm (prodigiosin-HNTs column) is the result of autofluorescence of prodigiosin released from prodigiosin-HNTs into the cytoplasm. Changes in nuclear morphology of Caco-2 and HCT 116 cells treated with p-HNTs shown in the far right column.

The changes of nanotubes adhesiveness after loading of prodigiosin affects their distribution over the cell surface (Figure 1D, red arrows). One can clearly distinguish relatively large amorphous aggregates of p-HNTs on cells (Figure 1D). HNTs *per se* distributed more evenly and less aggregate that was demonstrated in Supplementary Figure S4. The overall morphology of both HSF and MSC cells remains unaffected when cells interact with HNTs or p-HNTs, while some morphology changes observed in malignant cells (Figures 1D, 3 and Supplementary Figure S4). Internalization of p-HNTs and prodigiosin release from nanotubes started immediately after adding of nanotubes and increased in course of incubation time (Figure 2B). We observed the accumulation of prodigiosin in the perinuclear space as red fluorescent vesicles (Figures 2A,B). Prodigiosin leakage from loaded nanotubes into cytoplasm during cell cultivation may cause disorganization of the nucleus structure and reduce the volume of the cytoplasm in Caco-2 and HCT116 cells (Figures 1E, 4). In Caco-2 cells exposed to p-HNTs extracellular vesicles filled with prodigiosin can be observed (Figure 1E, yellow arrows). Noteworthy, we detected small vacuoles in the cytoplasm of p-HNTs treated fibroblasts (Figure 1E, green arrows). Vacuolization of HCT116 cells also was demonstrated after incubation of cells with p-HNTs for 3 h (Figure 2A). In Figure 2C the arrows indicate the absence of extracellular release of prodigiosin that can be seen in simultaneously taken dark-field and fluorescence images (red colored p-HNTs aggregate on the cell surface and cannot be visualized on fluorescence images).

**FIGURE 5:**
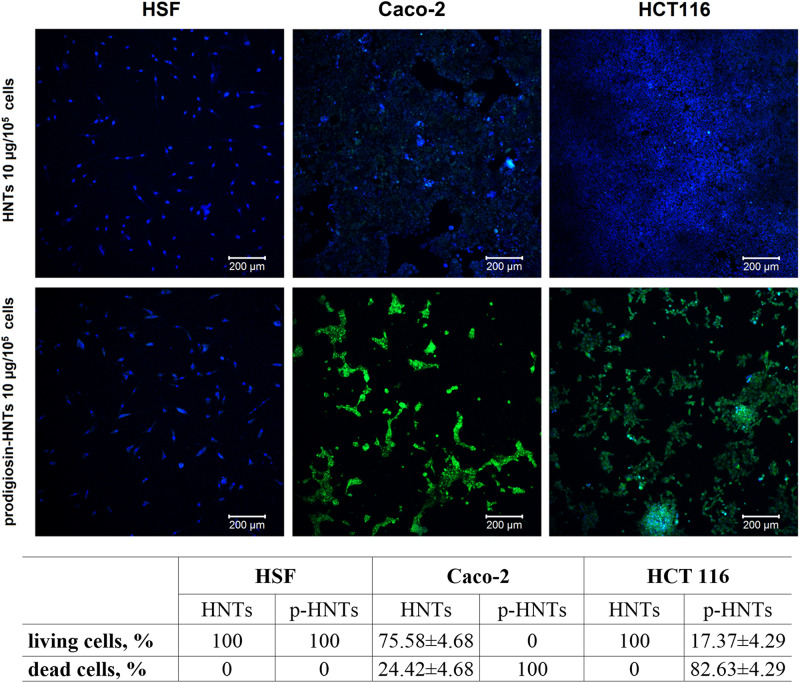
Live/Dead staining (green – dead cells, blue – living cells, Live/Dead kit (Life Technologies) of cells treated with HNTs and prodigiosin-HNTs (p-HNTs). Living cells appear blue and dead cells appearing green on the color-coded confocal images.

**FIGURE 6:**
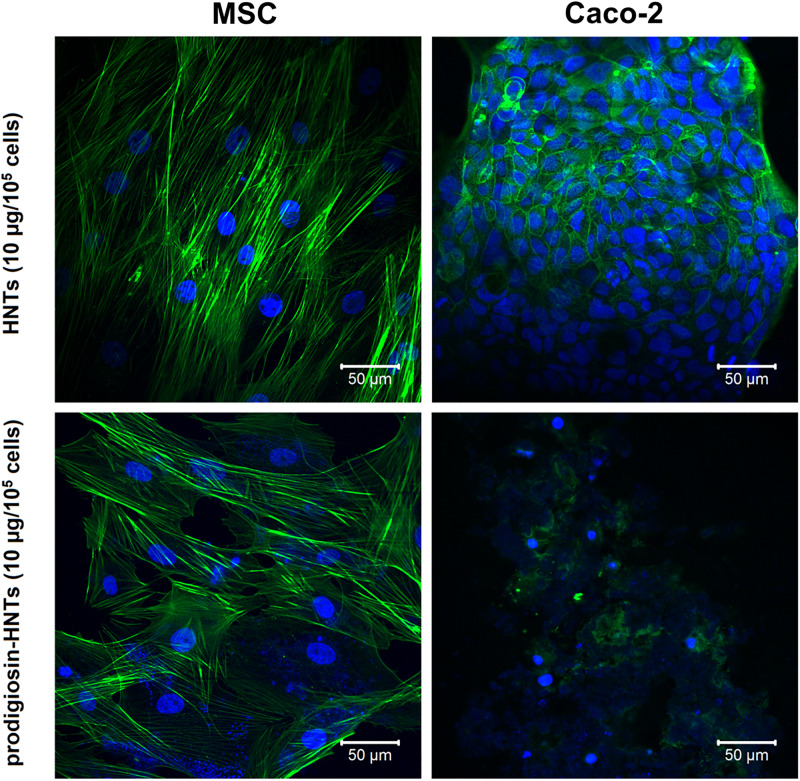
F-actin visualization in non-malignant (MSC) and malignant (Caco-2) cells after incubation with HNTs and prodigiosin-HNTs. Note the disorganization of F-actin after treatment of Caco-2 cells with prodigiosin-HNTs. MSC and Caco-2 cells where stained with DAPI (blue) and Alexa Fluor 488^®^ phalloidin (green). Figure shows a cytoskeleton disruption in cancer cells and the absence of such effect in MSCs (bottom images). Upper images clearly demonstrate that the occurrence of such effect is due to the prodigiosin action.

The addition of p-HNTs during the cultivation of cancer cells led to aberration of their morphology and subsequent detachment from the bottom of the dish (Figure 3). Glycerol-loaded HNTs used as a control did not affect the morphology and viability of all types of cells. The lack of long-term toxicity were observed for different type of test-organisms including yeast cells, Protista and worms (Konnova et al., 2013; Fakhrullina et al., 2015; Kryuchkova et al., 2016). The presence of p-HNTs in the culture medium of non-malignant cells led to increasing of vacuolization in some of them (Figure 3, inserts). However, fibroblasts and MSCs maintained normal morphology and viability as demonstrated using Live/Dead staining (Figure 5) where blue fluorescence indicates the nuclei of all cells while green stain is located exclusively in the nuclei of dead cells with compromised plasma membranes and cytoskeleton visualization (Figure 6). We summarized in Figure 5 the numerical data demonstrating the sensitivity of cells to HNTs and p-HNTs. Caco-2 cells were less resistant to HNTs in culture medium. The interaction with p-HNTs in concentration of 10μg per 10^5^ cells resulted to rapid 100% cell death. In opposite, the viability of non-malignant cells (HSF) did not change in all experimental variants. Considering the fact that prodigiosin can suppress cell proliferation, it is expected that the sensitivity of cancer cells to the inhibitory activity of prodigiosin depends on cell proliferation rates (Liu et al., 2018; Sam and Ghoreishi, 2018; Ji et al., 2019).

Cytoskeleton components are a known target for certain anticancer drugs (Yvon et al., 1999; Lin et al., 2016), which suppress microtubule dynamics, inhibiting, as a result, cell proliferation rates. Interestingly, treatment with prodigiosin demonstrates the same effects (Figure 6), apparently due to binding to cytoskeleton components followed by their structure disruption that in turn disrupts cytoskeleton functions. Thus, in this study we have found for the first time the changes in the morphology and viability of cancer cells and absence of such effect in non-malignant cells after exposure to prodigiosin-loaded halloysite nanotubes.

**FIGURE 7:**
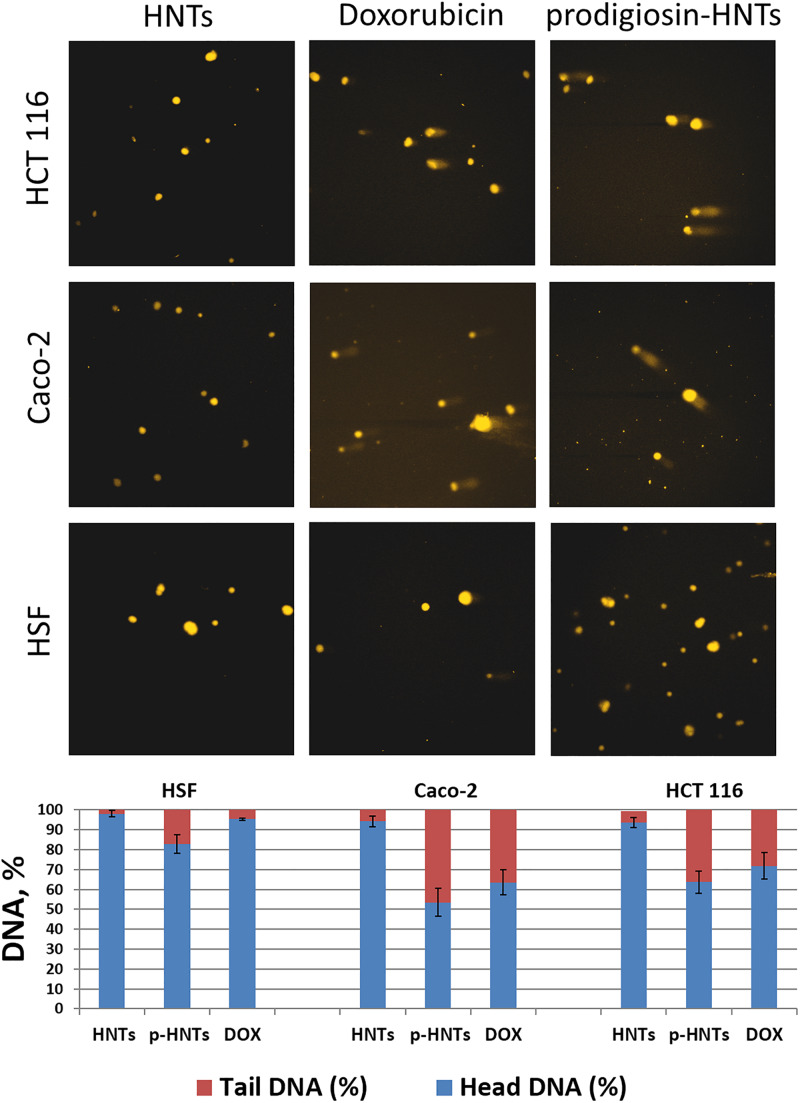
Genotoxic effects of prodigiosin-HNTs (p-HNTs) were evaluated using Comet Assay. Upper image – representative fields of view (fluorescent microscopy) with characteristic undamaged nucleoids and comets in each variant of experiment (specified in figure). The histogram shows the ratio of the DNA percentage in the tail and head of the comets. The semi-automated image analysis system Comet Score was used to evaluate 100 comets per sample.

To assess DNA damage caused by p-HNTs we used the Comet Assay or single-cell gel electrophoresis (Lorenzo et al., 2013) (Figure 7), which allows detecting DNA degradation in individual cells. Briefly, cells were embedded in agarose gel and distributed on adhesive microscope slide, then cells were lysed, leaving nucleoids (DNA structures without nuclear membrane), and then electrophoresed in alkali conditions. DNA with strand breaks are relaxed and extend toward the anode during the electrophoresis, forming a comet-like tail viewed by fluorescence microscopy with ethidium bromide staining. DNA damage (strand breaks frequency) is related to the percentage of DNA in the tail. Undamaged DNA remains in the place of initial cell localization and represent the head of comet-like structure. Anticancer drug doxorubicin was used as a positive control. Prodigiosin can bind with DNA by intercalation and acts mainly as inhibitors of topoisomerases I and II (Lins et al., 2015). This effect can cause DNA damage, which is directly correlated with the level of cytotoxicity and genotoxicity of p-HNTs in our study. We found that genotoxic effect of p-HNTs studied by Comet Assay was more pronounced in the case of malignant cells (Caco-2, HCT116). The formation of strand breaks of DNA was observed with much less frequency in human skin fibroblasts treated with p-HNTs. HNTs without prodigiosin caused only background level of DNA damage in all cell types, therefore we assume that the possible reason for p-HNTs selective antitumor action might be the destruction of malignant cells DNA.

## Conclusion

In summary, prodigiosin was successfully adsorbed by HNTs surfaces and encapsulated within halloysite lumen. Then, we found that prodigiosin could release in the cytoplasm of cells, while no release occurs extracellularly. *In vitro* anticancer effects of p-HNTs were manifested in the suppression of Caco-2 and HCT116 cells proliferation, followed by alteration of cell morphology and F-actin structure disorganization. Comet assay response where fragmented chromatin were observed, indicating a high therapeutic effect of halloysite formulated prodigiosin. Comparison of the effects of p-HNTs on malignant (Caco-2, HCT116) and non-malignant (MSC, HSF) cells allows to conclude that the p-HNTs demonstrate the selective cytotoxic and genotoxic activity. We hypothesize that prodigiosin entrapped into halloysite may have significant advantages for treatment of living tissues *in vivo*, due to higher bioavailability and extended intracellular release.

## Data Availability Statement

The datasets generated for this study are available on request to the corresponding author.

## Author Contributions

IG, EN, and RF designed the research. IG and EN performed the cell culture studies. FA performed the AFM experiments. GL and GC performed the thermoanalysis. LN and EN performed the dark-field microscopy experiments. All the authors read and approved the final version of the manuscript.

## Conflict of Interest

The authors declare that the research was conducted in the absence of any commercial or financial relationships that could be construed as a potential conflict of interest.
