# Supplementary material for: Selective Cytotoxic Activity of Prodigiosin@halloysite Nanoformulation
Source: Front Bioeng Biotechnol. 2020 May 26;8:424. doi: 10.3389/fbioe.2020.00424 (PMC7264093; doi:10.3389/fbioe.2020.00424)
Supplement: Supplementary file 1 [file Image_1.pdf]

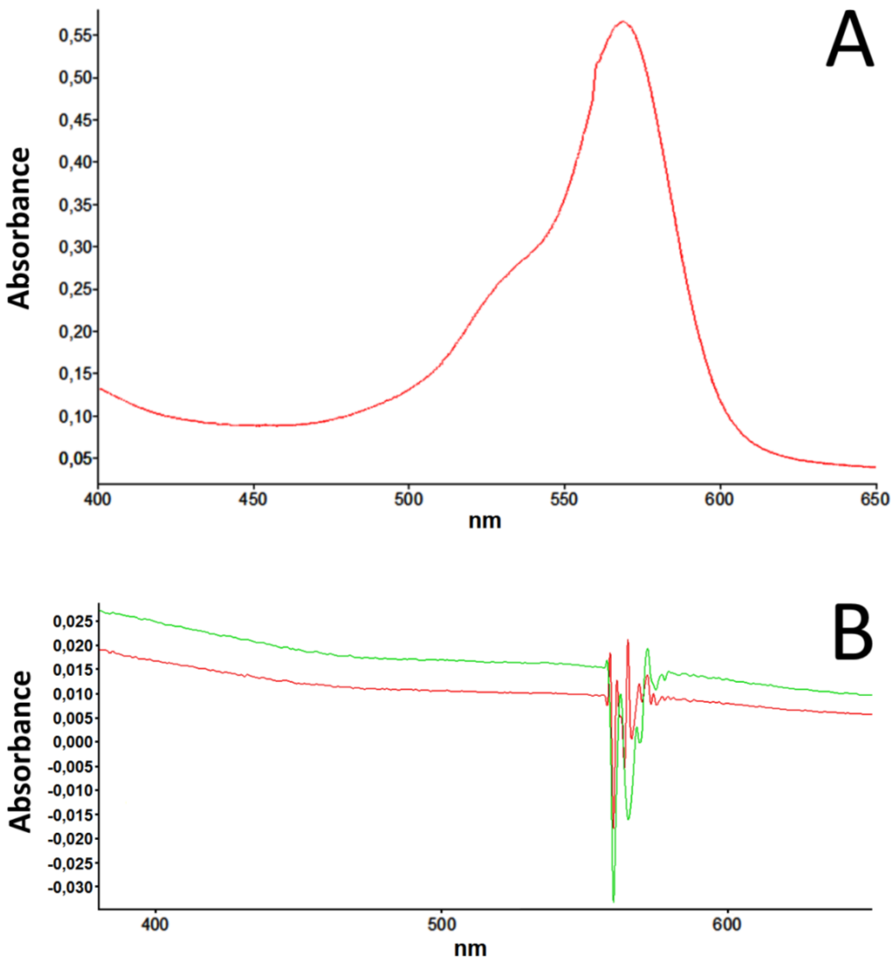

Figure S1. Absorption spectra of ethanol solution of prodigiosin **(A)** and release from loaded halloysite nanotubes **(B)**. Absorption spectra of prodigiosin-loaded halloysite nanotubes suspension in PBS after 30 min (red curve) and 2 h (green curve) of exposure demonstrate the absence of prodigiosin leakage in extracellular conditions.

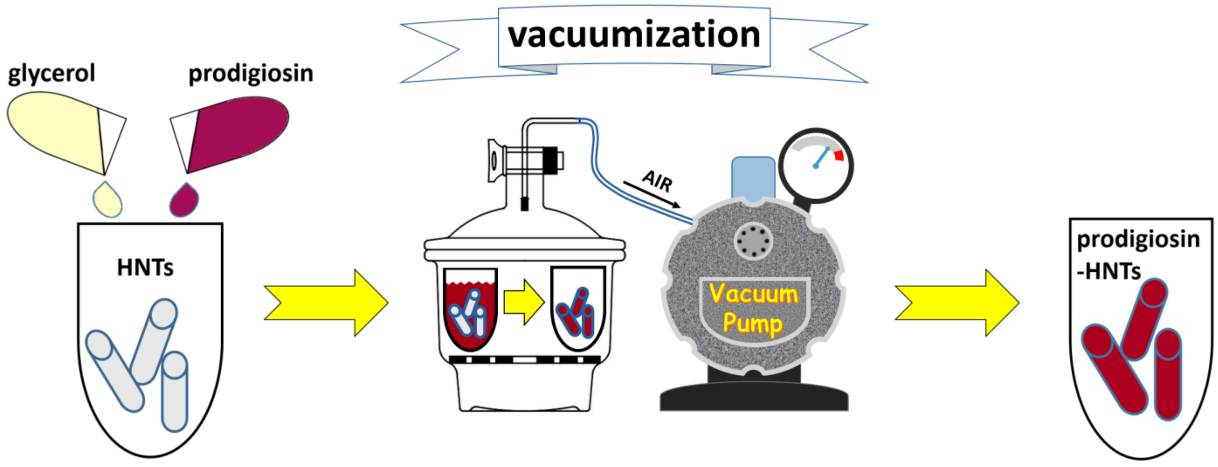

Figure S2. Prodigiosin-HNTs fabrication. Ethanol solution of prodigiosin (96% vol.) was mixed with glycerol and dry HNTs in centrifuge tube then placed into desiccator for loading by vacuumization.

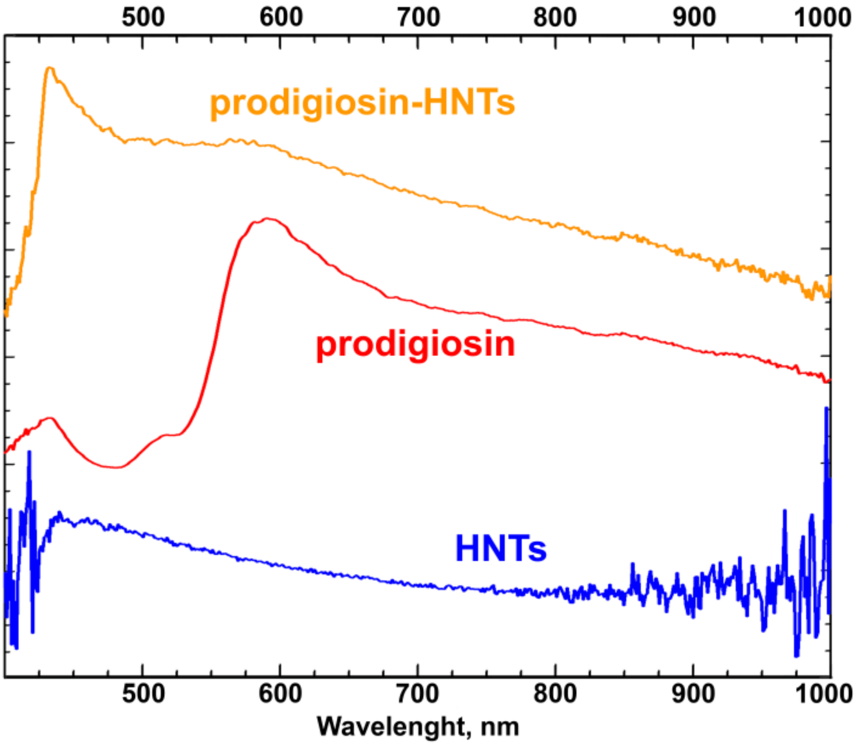

Figure S3. Spectral profiles of pure halloysite nanotubes (HNTs), prodigiosin and prodigiosin-loaded halloysite nanotubes (prodigiosin-HNTs).

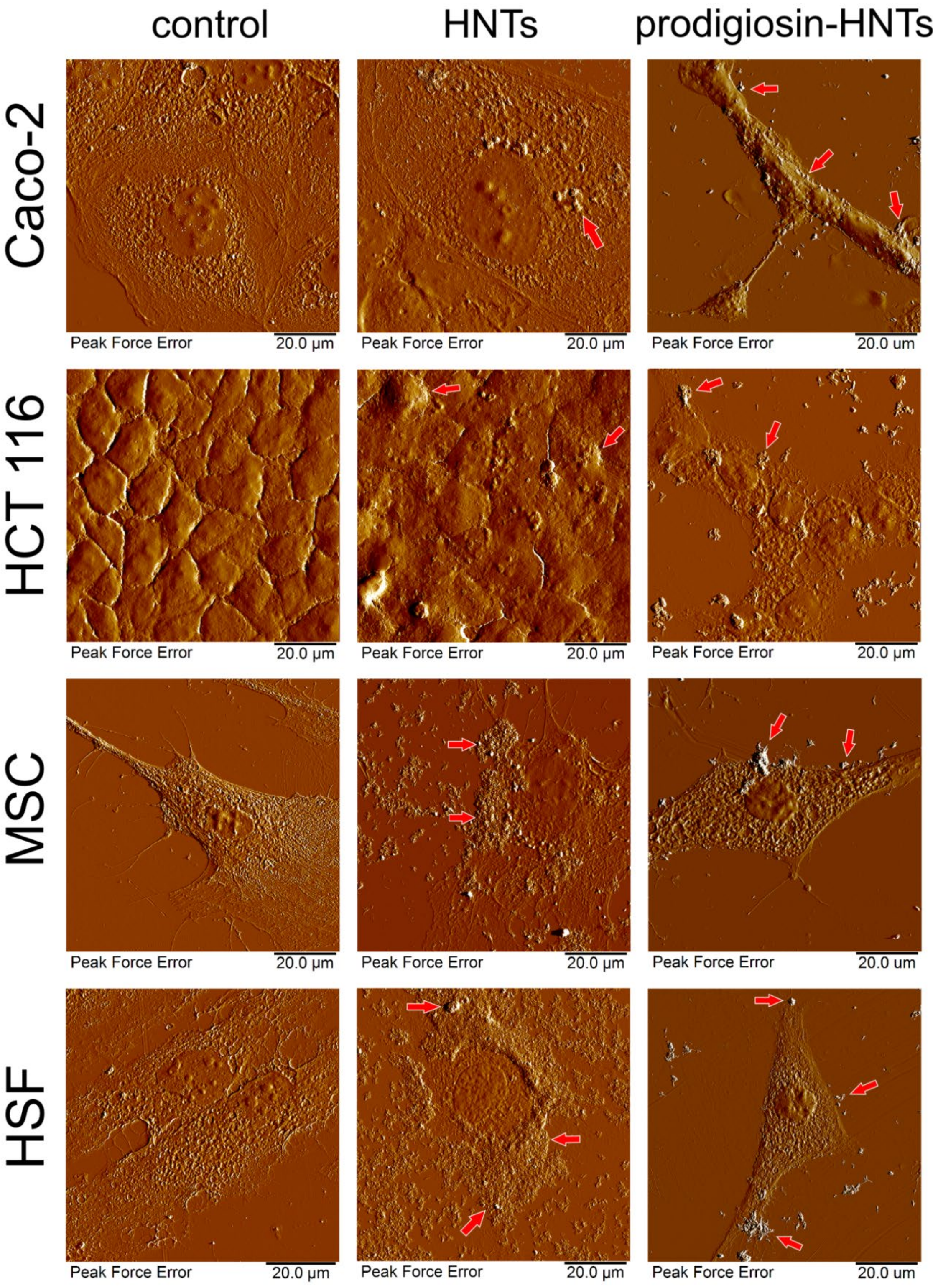

Figure S4. AFM images (Peak Force Error mode) demonstrate morphology and surface structure of the intact cells (control) and cells after treatment with HNTs/prodigiosin-HNTs. Prodigiosin-induced morphological changes of cells and cell monolayers were expressed more brightly in cancer cells (Caco-2 and HCT-116). Red arrows mark the location of nanotubes clusters. More condensed clusters of prodigiosin-HNTs possibly resulted due to highly adhesive activity of prodigiosin-loaded halloysite nanotubes. The decrease of cell index that was observed after interaction of malignant cells with prodigiosin-HNTs may be associated with the ability of prodigiosin to induce the disorganization of actin cytoskeleton in cancer cells and cause selective DNA damage.
